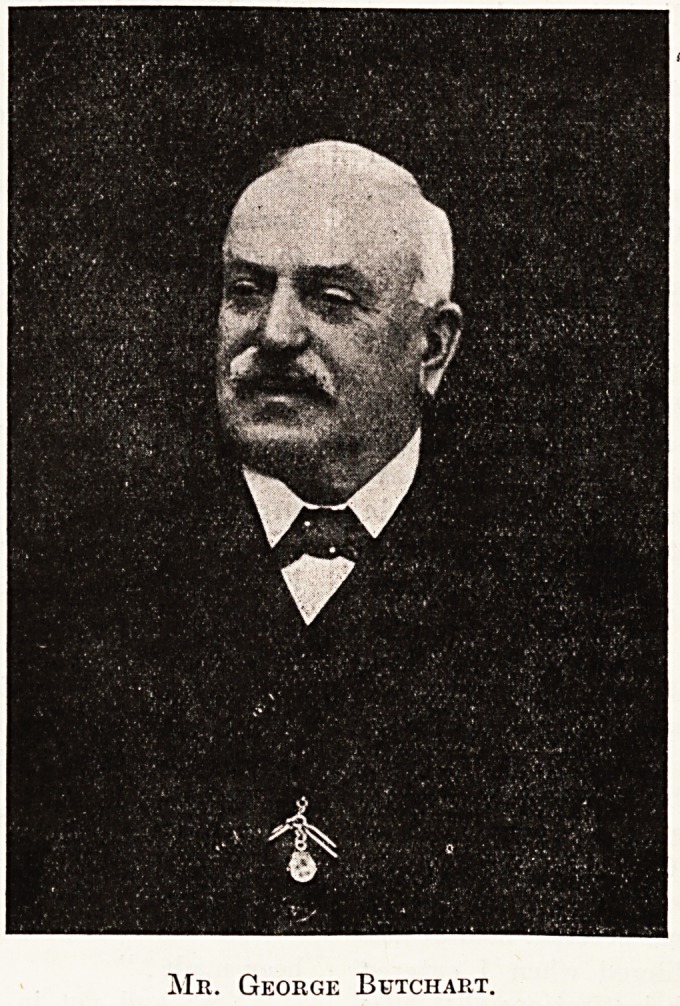# Mr. George Butchart, Chairman of the House Committee

**Published:** 1913-03-29

**Authors:** 


### EMINENT CHAIRMAN SERIES.

### Mr. George Butchart, Chairman of the House Committee,

THE DURHAM COUNTY AND SUNDERLAND EYE INFIRMARY.

THE Durham County and Sunderland Eye Infirmary has always been fortunate in having as its chairmen gentlemen of wide experience and much enthusiasm. Indeed, the very satisfactory financial position of the institution and its popularity are largely due to the self-sacrificing labours of the late Dr. Hopgood, who was honorary surgeon for thirty-five years and chairman of the general committee from 1894 until his death in 1909. That this prosperity will be maintained the personality of Mr. G. O. Wight, J.P., the present chairman of the general committee, is a sufficient guarantee, for Mr. Wight's knowledge of finance and the great interest he takes in the Eye Infirmary are well known to all Sunderland residents, and to the working-men of the locality, whose subscriptions form the mainstay of the institution.

In the present article, however, we are chiefly concerned with the scientific and general equipment of the institution, and with the Chairman of the House Committee, Mr. George Butchart, who has worked very hard to render them efficient and up to date. Mr. George Butchart first became a member of the general committee in 1889, and in 1893 was elected Chairman of the first House Committee to be formed after the Eye Infirmary was removed to Stockton Road. He has, therefore, completed almost twenty years of work in the latter capacity, the nature and value of which can only be appreciated by those who have come closely into touch with the institution and its administrative head.

MR. GEORGE BUTCHART.

The Durham County and Sunderland Eye Infirmary was founded in 1836, when the work was carried on for many years in a single room over a shop in one of the poorest quarters of the town. In 1881 a single dwelling-house was purchased in Crowtree Road for the purposes of the institution, and for the first time the surgeons had beds, to the number of five, at their disposal. But their usefulness was largely discounted by the fact that the poor in-patients had to provide their own food. The number of out-patients treated in this building in 1887 was just over 1,000, so that the progress made in fifty years, to say the least, was very gradual.

The modern development of the institution may be dated from 1893, when it was removed to a specially designed building in Stockton Road. Since that time progress has been continued, as may be seen by a great advance in the number of patients treated, the astonishing increase in the working-men's financial support, and a high standard of scientific efficiency.

After sixteen years—that is, in 1909—it was found that the accommodation and equipment of the Eye Infirmary again required enlarging and bringing up to date. The carrying out of this work will long remain a monument to the indefatigable energy and devotion of Mr. George Butchart. It was his determination that no stone should be left unturned to render the institution efficient in every respect. He visited other hospitals that had recently been modernised in order to see for himself the working of various innovations. He made himself conversant with modern hospital construction and surgical requirements, and he consulted the wishes of the surgical staff at every turn.

Not content with passing plans and supervising estimates, Mr. Butchart actually superintended every detail of the work himself. His remarkable knowledge of building construction ensured that nothing but the best materials and workmanship were put into the contract.

As a mark of their appreciation of the continuous and unceasing time and thought devoted by Mr. Butchart to this work, the members of the general committee subscribed for a handsome silver rose-bowl, and this was presented to him at the opening of the enlarged out-patient department in September 1911.

The efficiency of the general arrangements of this hospital to-day have been brought about by the Chairman of the House Committee, to whom the institution owes a debt of gratitude not easily exaggerated. But a hospital, however up to date it may be, is of little use unless a spirit of *esprit de corps* prevails amongst its workers. Such a spirit is very strongly present in the Sunderland Eye Infirmary, and is due in no small measure to the kindness and consideration with which Mr Butchart watches over the welfare and interests of all grades of its workers. We may refer readers to our Report on the Hospital which is alluded to in our Notes this week.

## Figures and Tables

**Figure f1:**